# The Influence of Chitosan Cross-linking on the Properties of Alginate Microparticles with Metformin Hydrochloride—In Vitro and In Vivo Evaluation

**DOI:** 10.3390/molecules22010182

**Published:** 2017-01-22

**Authors:** Marta Szekalska, Katarzyna Sosnowska, Agnieszka Zakrzeska, Irena Kasacka, Alicja Lewandowska, Katarzyna Winnicka

**Affiliations:** 1 Department of Pharmaceutical Technology, Medical University of Białystok, Mickiewicza 2c, Białystok 15-222, Poland; marta.szekalska@umb.edu.pl (M.S.); katarzyna.sosnowska@umb.edu.pl (K.S.); 2 Department of Biopharmacy, Medical University of Białystok, Mickiewicza 2c, Białystok 15-222, Poland; azakrze@umb.edu.pl; 3 Department of Histology and Cytophysiology, Medical University of Białystok, Mickiewicza 2c, Białystok 15-222, Poland; kasacka@umb.edu.pl (I.K.); alicja.lewandowska@umb.edu.pl (A.L.)

**Keywords:** alginate, chitosan, cross-linking, microparticles, spray drying, hypoglycemic activity, metformin hydrochloride, toxicity

## Abstract

Sodium alginate is a polymer with unique ability to gel with different cross-linking agents in result of ionic and electrostatic interactions. Chitosan cross-linked alginate provides improvement of swelling and mucoadhesive properties and might be used to design sustained release dosage forms. Therefore, the aim of this research was to develop and evaluate possibility of preparing chitosan cross-linked alginate microparticles containing metformin hydrochloride by the spray-drying method. In addition, influence of cross-linking agent on the properties of microparticles was evaluated. Formulation of microparticles prepared by the spray drying of 2% alginate solution cross-linked by 0.1% chitosan was characterized by good mucoadhesive properties, high drug loading and prolonged metformin hydrochloride release. It was shown that designed microparticles reduced rat glucose blood level, delayed absorption of metformin hydrochloride and provided stable plasma drug concentration. Additionally, histopathological studies of pancreas, liver and kidneys indicated that all prepared microparticles improved degenerative changes in organs of diabetic rats. Moreover, no toxicity effect and no changes in rats behavior after oral administration of chitosan cross-linked alginate microparticles were noted.

## 1. Introduction

Sodium alginate (ALG) belongs to the group of polysaccharides naturally produced by seaweeds and bacteria [1,2]. It is nontoxic, biocompatible and biodegradable polymer, which is composed of β-d-mannuronic acid and α-l-guluronic acid residues joined by (1–4) glycosidic linkages. ALG is characterized by swelling and mucoadhesive properties and possesses unique ability to gel [3]. Common used gelling method is ionic cross-linking process, which is known as “egg-box” model. Mechanism of this process includes binding of divalent or trivalent cations by the guluronate blocks of the polymer chain which provides gel forming. Calcium chloride (CaCl_2_) is one of the most frequently used ALG ionically cross-linking agents [3,4].

As ALG is an anionic polymer, it exhibits physical properties to electrostatic interaction with oppositely charged polymers such as chitosan and, consequently, it forms polyelectrolyte complexes [5]. In result of ionic interaction, intramolecular bonds between ALG carboxylic acid groups and the protonated amine groups of chitosan are created [6,7]. Chitosan is a linear polysaccharide consisting of glucosamine and *N*-acetylglucosamine units linked by β-1,4 glycosidic bonds, obtained by partial deacetylation of chitin. It is a biocompatible, biodegradable and nontoxic polymer [8,9]. ALG connection with chitosan may be used to exploit the specific properties the individual polymers and to minimize their disadvantages. It was confirmed that ALG-chitosan polyelectrolyte complex is characterized by improved thermal, chemical and mechanical stability of constituent polymers. Additionally, drug dosage forms based on the ALG-chitosan complexes are characterized by the higher swelling degree, mucoadhesive force and prolonged drug release [10,11]. Improvement of mucoadhesive properties of polymer is an important aspect in the design of sustained release dosage forms [12,13].

Metformin hydrochloride (MF) is an antidiabetic agent from the biguanide group, commonly used in noninsulin-dependent diabetes mellitus. Mechanism of hypoglycemic activity of MF is based on the hepatic inhibition of glucose production and intestinal glucose absorption. MF after oral administration is characterized by short biological half life (2.5–3 h), incompletely absorption and bioavailability about 50% [14,15,16]. Mucoadhesive microparticles are an example of multiunit systems, which ensure intimate contact with mucus layer and provide efficient absorption and enhanced bioavailability of drugs [17,18]. The commonly used methods to obtain ALG microparticles using chitosan as cross-linking agent are coacervation, emulsification/solidification, and solvent extrusion, which have many disadvantages such as the difficulty in controlling the process, the use of organic solvents and low drug encapsulation [19,20,21]. Spray-drying is a technique widely used in many industries, e.g., food, pharmaceutical, chemical and cosmetic. This technique can be used for the microencapsulation of hydrophilic or hydrophobic and thermolabile active substances. In contrast to conventional methods, spray-drying is a single step, rapid, continuous, cost-effective and scalable process with high versatility for the production of dry powders. Spray drying is one of the most reproducible techniques, enables precisely control of the process parameters, allows obtaining a product with the desired properties, and can be used at both laboratory and industrial scale. Spray dried microparticles are usually characterized by spherical shape and narrow particle size distribution. Additionally, spray-drying technique provides to obtain microparticles with relatively high production yield and encapsulation efficiency [22,23,24]. 

In our earlier work it was observed that ALG microspheres with MF prepared by the spray-drying method possessed good swelling and mucoadhesive properties, but the drug release profile was prolonged only up to 4 h [25]. Therefore, in order to obtain more sustained drug release, developing and evaluating for the first time, the possibility of preparing chitosan cross-linked ALG microparticles containing MF by the spray-drying technique was examined. In addition, influence of cross-linking agent on the properties of microparticles was evaluated. Formulated cross-linked microparticles were characterized in terms: size, morphology, entrapment efficiency, drug loading, Zeta potential and the in vitro MF release. Swelling ratio and mucoadhesive properties were also examined. The physical state of microparticles was determined by differential scanning calorimetry (DSC). Additionally, ALG effect on the hypoglycemic MF activity, MF plasma concentration and its pharmacokinetic parameters were examined in vivo in diabetic rat model. To evaluate potential toxic activity of designed microparticles, the pancreas, liver and kidneys morphology were examined.

## 2. Results and Discussion

### 2.1. Microparticles Morphology, Size and Surface Charge Analysis

**Figure 1:**
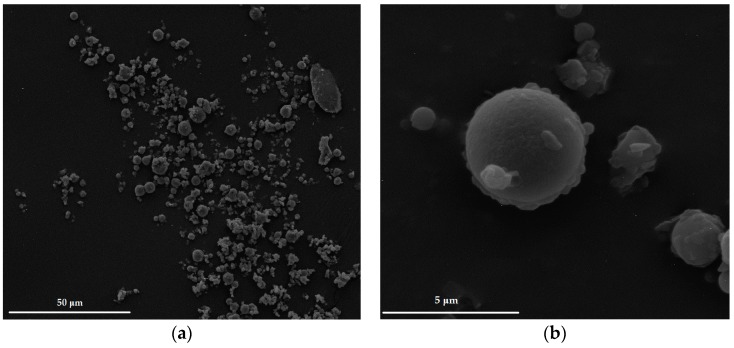
Representative images of microparticles formulation CH1M under magnification: (**a**) ×2000; and (**b**) ×20,000.

The morphology of formulated microparticles is presented in Figure 1. The obtained results have shown that one-step spray-drying process might be successfully used to prepare microparticles using 2% of ALG solution and chitosan as cross-linking agent. It was observed that the most part of prepared microparticles were characterized by spherical shape with little rough area. Interestingly, cross-linking process did not affect the mean microparticles diameter (Table 1). Polydispersity index was in the range from 0.38 ± 0.1 to 0.52 ± 0.2. The obtained data suggest that the fluctuation in the particle size was cross-linking agent concentration dependent.

The concentration of cross-linking agent had also significant impact on production yield. When chitosan concentration was increased, production yield was decreased (from 85.9% ± 1.4% to 61.8% ± 1.5% in CH2M and CH1M, respectively), which could be a result of increase in the viscosity of the solution. Simultaneously, it was observed that concentration of cross-linking agent did not affect encapsulation efficiency.

Zeta potential is a parameter which enables predicting the stability of formulations [26]. ALG is an anionic polymer with negative charge, and chitosan as cross-linking agent changed Zeta potential [25,27] from negative (−1.3 ± 0.7 mV) to positive values (6.7 ± 1.3 mV for CH1M and 5.2 ± 0.3 mV for CH2M).

### 2.2. Swelling and Mucoadhesive Properties

**Figure 2:**
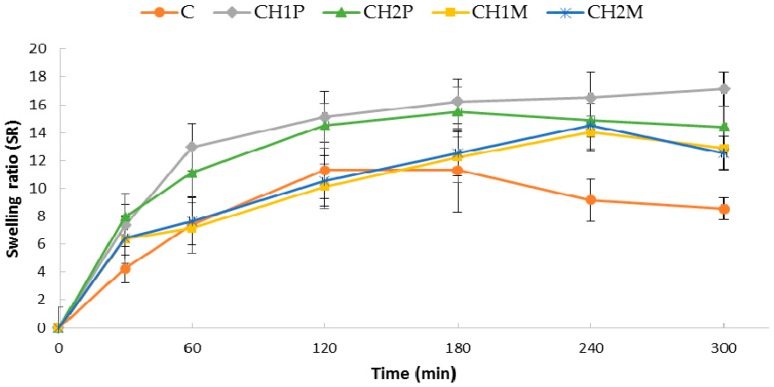
Swelling ratio (SR) of non cross-linked microparticles (C) and cross-linked microparticles CH1P, CH2P, CH1M, and CH2M (*n* = 3).

Swelling of polymer is a key factor in mucoadhesion process and drug release [28]. The swelling properties evaluated in 0.1 M HCl (pH 1.2) were expressed as the swelling ratio (SR) at different time intervals, as shown in Figure 2. It was observed that all obtained curves (except formulation C) gave linear increase for 180 min of the study, which indicates that all microparticles were characterized by significant swelling ability. It was found that chitosan cross-linked ALG microparticles were characterized by higher swelling properties compared to the non cross-linked microparticles formulation C. Formulations CH1P and CH2P exhibited the highest SR values (17.1 ± 1.4 after 300 min and 15.5 ± 1.8 after 180 min of the study, respectively) (Figure 2). SR of designed microparticles was chitosan concentration dependent: higher chitosan content in microparticles prevented the shrinking of ALG and provided higher swelling properties. Additionally, it was observed that microparticles formulation CH1M and CH2M were characterized by lower SR, which was related with the presence of freely soluble in acidic environment MF facilitating water penetration into microparticles matrix.

Swelling is the first stage of mucoadhesion process and in the presence of moisture, swelling polymer initiates contact with the mucus layer, leads to mechanical entanglement and, consequently, to the formation of hydrogen bonds and/or electrostatic interactions between the polymer and the mucus network [29,30,31]. Carboxyl and hydroxyl groups of ALG are responsible for the formation of hydrogen-bonds between the polymer and the mucus layer, but introducing additional components into the microparticles can affect the adhesion strength [32]. The mucoadhesive properties, presented as maximum detachment force (F_max_) and work of adhesion (W_ad_), are shown in Table 2. It was noticed that all designed microparticles adhered to all tested mucoadhesive materials and that detachment force and work of adhesion was dependent on the type of adhesive layer and cross-linking agent concentration. When porcine stomach mucosa was used as adhesive layer, the highest values of the F_max_ and W_ad_ were observed. The highest values of F_max_ (1.4 ± 0.2 N) and W_ad_ (782.6 ± 29.2 μJ) were observed for cross-linked formulation CH1P (with higher concentration of chitosan).

### 2.3. In Vitro MF Release

**Figure 3:**
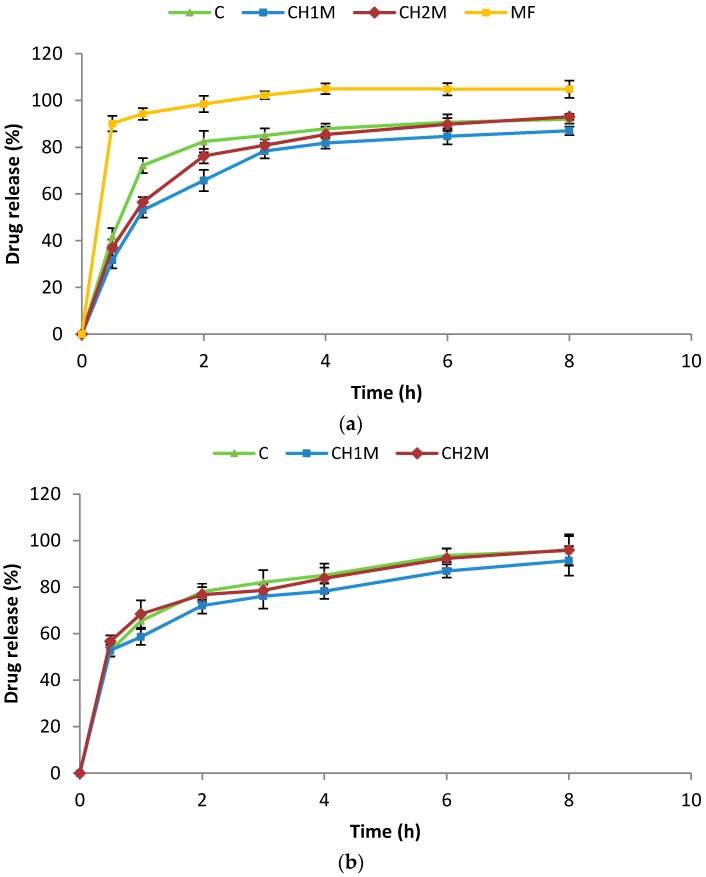
MF release from non cross-linked microparticles formulations C, cross-linked microparticles formulations CH1M, CH2M and non-modified commercially available tablet with metformin hydrochloride (MF): (**a**) after preparation; and (**b**) after six-month storage at 40 ± 2 °C and 75% ± 5% RH (*n* = 3).

MF release from non cross-linked microparticles formulations C and cross-linked microparticles formulations CH1M and CH2M is presented in Figure 3. It was observed that release profiles in all formulations were characterized by burst effect, which may be related with the high water soluble nature of free MF present at the microparticles surface. ALG in contact with acidic pH is converted to swelling and gelling alginic acid, which controls water penetration inside the microparticles structure. The results of dissolution studies indicated that the microparticles formulations CH1M and CH2M showed prolonged release (up to 6 h) compared to formulation C. Even though chitosan is soluble in acidic environment, its use as cross-linking agent in ALG microparticles cause the change in the matrix properties through increased of microparticles swelling properties and MF release was more sustained [13,33].

MF release profiles from all formulations were examined according to zero-order and first-order equations and Higuchi, Korsmeyer–Peppas, and Hixson–Crowell models (Table 3). The obtained data indicated that mechanism of MF release from all formulations was based on the first-order kinetics, where the plots showed the highest regression correlation coefficient. These results were confirmed by Highuchi model, which was characterized by the high values of R^2^. Moreover, in case of Korsmeyer–Peppas model, value of diffusional exponent *n* was in the range from 0.11 to 0.19, which also indicated that mechanism of MF release was diffusion dependent. Hixson–Crowell plot showed good linearity with regression value from 0.93 to 0.94 in chitosan cross-linked ALG microparticles indicating mechanism of MF release is combination of diffusion and erosion determined by cross-linking agent [34,35].

### 2.4. Stability Studies

No significant changes in physical appearance, mean diameter, drug loading, Zeta potential values, mucoadhesive properties and drug release profiles from non cross-linked microparticles formulation C and cross-linked microparticles formulations CH1M and CH2M stored six months at 25 ± 2 °C and 60% ± 5% RH were observed (data not shown). After six months of storage at 40 ± 2 °C and RH 75% ± 5%, only minor changes in particle size, Zeta potential values and mucoadhesive properties with no appreciable change in drug content and drug release profiles were noted (Figure 3b, Table 4).

### 2.5. Differential Scanning Calorimetry (DSC)

**Figure 4:**
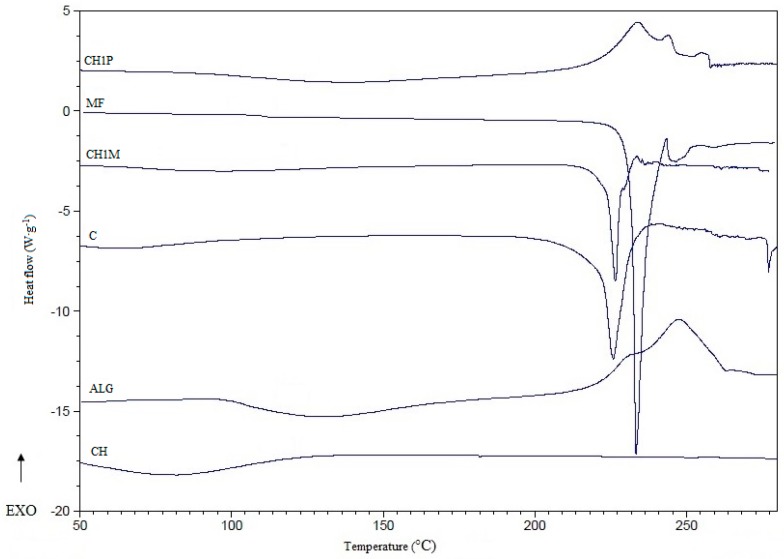
DSC thermograms of sodium alginate (ALG), pure metformin hydrochloride (MF), non cross-linked microparticles formulation C, chitosan (CH), cross-linked microparticles placebo (CH1P) and cross-linked microparticles formulation CH1M.

Differential scanning calorimetry is a useful technique to characterize the thermal behavior of materials. DSC thermograms provide information about possible interactions between drug and polymer through the analysis of parameters such as shift, appearance and disappearance of peaks, melting point and relative peak area [36]. Thermograms of the non cross-linked microparticles formulation C, cross-linked microparticles formulation CH1M, and their components are shown in Figure 4. Sharp endothermic peak for pure MF corresponding to its melting point was observed at 233 °C. The thermogram of pure chitosan showed a broad endothermic peak between 50 °C and 100 °C in consequence of the water loss of polymer. In thermogram of microparticles formulation CH1P, broader peak in the range from 220 °C to 255 °C was observed with additional signals, which might suggest an interaction between ALG and chitosan [37,38]. Moreover, the peaks corresponding to ALG and chitosan disappeared in the thermogram of drug loaded formulation, which is characteristic for water loss from the sample [38]. DSC peak of MF showed no significant decrease of melting temperature in microparticles formulation CH1M (226 °C) compared to the pure drug (233 °C) [36,37,38].

### 2.6. In Vivo Studies

In this study, diabetes in rats was induced according to the Zhang [39]. The obtained data of glucose plasma concentration in rats from the control group during the 28 days of experiment indicated that high-fat diet combined with double low doses of streptozotocin (30 mg/kg b.wt. at biweekly intervals) was a good model for developing a stable animal model of type 2 diabetes.

#### 2.6.1. Blood Glucose Levels

**Figure 5:**
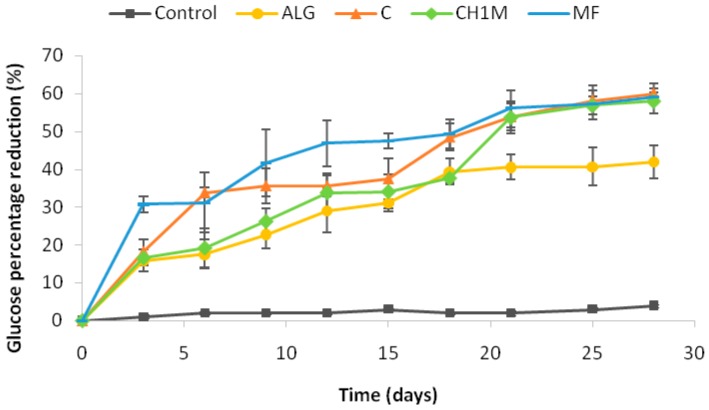
Percentage reduction in blood glucose level in rats treated with carboxymethyl cellulose sodium salt (Control), microparticles placebo (ALG), non cross-linked microparticles formulation C, cross-linked microparticles formulation CH1M and commercially available tablet with metformin hydrochloride (MF) after 28 days treatment (*n* = 6).

The effect of orally administrated non cross-linked ALG microparticles formulation C, chitosan cross-linked ALG microparticles formulation CH1M and commercially available tablet with MF on changes in plasma glucose in the streptozotocin-induced diabetic rats is presented in Figure 5 as percentage reduction of glucose level. The reduction of blood glucose concentration in rats treated with microparticles formulation C and CH1M was slower than in rats treated with MF tablets, which was related with lower and more sustained drug plasma concentrations. Gradual reduction of blood glucose level was observed and, after 21 days, it achieved similar values to MF tablet. Additionally, the obtained data confirmed hypoglycemic activity of pure ALG, which is promising hypoglycemic agent in obesity and type 2 diabetes treatment [40,41]. In rats treated with ALG microparticles placebo, 15% and 33% reduction in blood glucose was observed after 3 and 18 days of treatment, respectively.

#### 2.6.2. Pharmacokinetic Parameters

**Figure 6:**
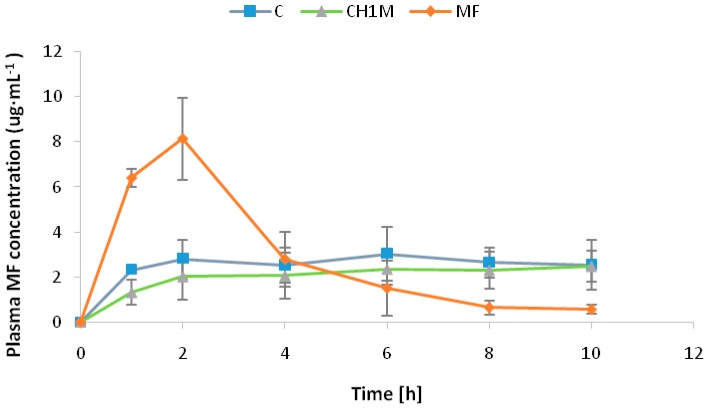
Profiles of metformin hydrochloride plasma concentration after single oral administration of non cross-linked microparticles formulation (C), cross-linked microparticles formulation (CH1M) and commercially available tablet with metformin hydrochloride (MF) in an amount corresponding to the 100 mg/kg b.wt. (*n* = 6).

The plasma concentration profile and pharmacokinetic parameters of MF after oral administration of non cross-linked microparticles formulations C, cross-linked microparticles formulations CH1M and commercially available MF tablet are shown in Figure 6 and Table 5. It was observed that MF absorption from the conventional tablets was rapid as indicated by low t_max_ value (2 h) in comparison to microparticles formulations C and CH1M, which exhibited delayed absorption as demonstrated by higher t_max_ (6 h) values. C_max_ value of conventional tablet was 8.13 ± 0.50 μg/mL compared with microparticles formulations C and CH1M: 3.03 ± 0.70 μg/mL and 2.37 ± 0.38 μg/mL, respectively. The obtained data suggest that administration of microparticles formulation C and CH1M provides a stable plasma drug level.

#### 2.6.3. Histopathological Studies

**Figure 7:**
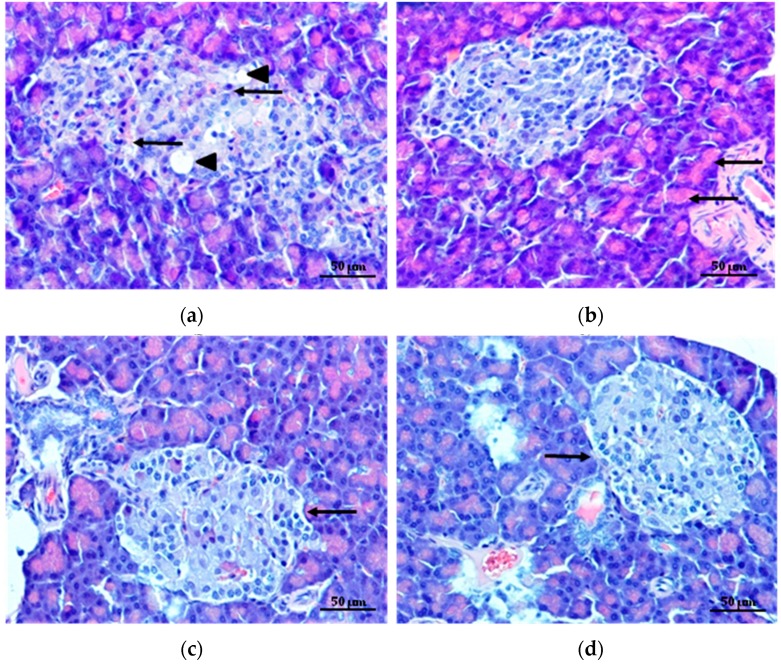
Representative hematoxylin and eosin stained paraffin pancreas section of diabetic rats (**a**) shows in the endocrine part the islets tended to be enlarged and irregular in shape. Throughout the island, large amount of eosinophilic cells (arrows) and fat droplets (arrowheads) are seen. Pancreas of diabetic rats treated with MF (**b**) depicts islet of Langerhans with a regular shape, surrounded by strongly stained acinar cells. The distant (from the lumen) parts of the glandular cells show basophilic granules and they are stained more deeply than the inner parts (arrows). Pancreas of diabetic rats treated with non cross-linked microparticles formulation C (**c**) depicts normal organ structure with significant reduction of necrosis and degeneration (arrow). Pancreas of diabetic rats treated with cross-linked microparticles formulation CH1M (**d**) shows improvement of irregular shape of Langerhans islets with reduction of degenerative changes (arrow). Magnification ×200.

Although ALG and chitosan are known as biocompatible and biodegradable polymers [2,42,43], reports of the toxicity of microparticles prepared with pure and modified by cross-linking polymers by the spray drying is limited. Pancreas, liver and kidney are organs, which are the most exposed to damage [44], therefore these organs were selected to histopathological studies. Effects of microparticles on the pancreas morphology and representative images of pancreatic islets are shown in Figure 7a–d. Microscopic examination of pancreatic sections of diabetic rats revealed histopathological changes, such as congestion in blood vessels of the gland, the fat droplets and disorders in Langerhans islets structure. In addition, a slight vacuolization, clear unstained areas, especially around the nucleus and strong eosinophilic cytoplasm of endocrine cells were observed (Figure 7a). The histopathological studies of pancreas indicated also that the long-term high fat diet together with double streptozotocin injection in low dose 30 mg/kg b.wt. provided degeneration of the Langerhans islets with fat deposition (Figure 7a), which might be successfully used to simulate human type 2 diabetes and is suitable model for examining the histopathological effects in the organs [45]. Pathological changes in the pancreas were alleviated in diabetic rats treated with MF (Figure 7b). Light microscopic analysis of pancreas from diabetic rats treated with non cross-linked microparticles formulation C and cross-linked microparticles formulation CH1M showed typical, oval or circular shapes of islets without pathological changes. Additionally, decrease in number of cells with strongly eosinophilic cytoplasm and only single cells with vacuolization were noted (Figure 7c,d).

**Figure 8:**
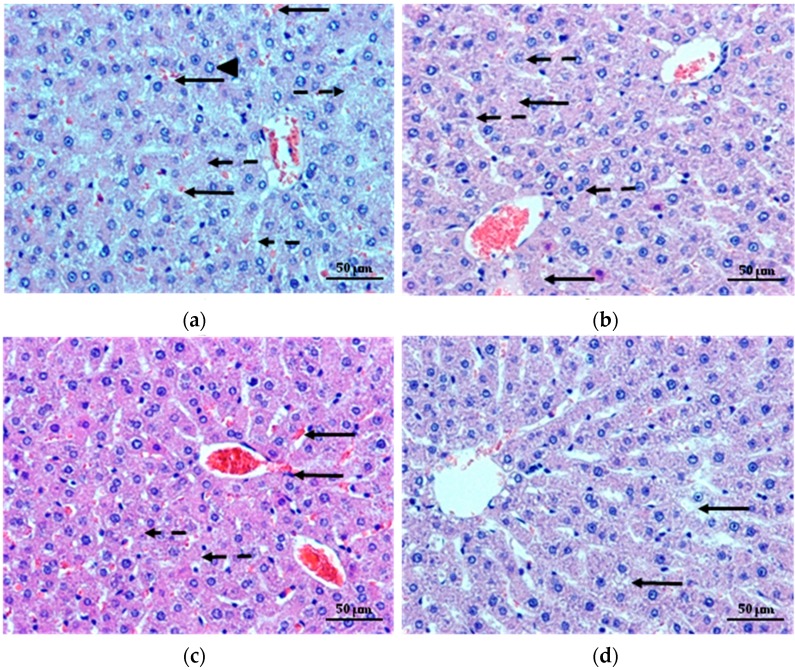
Representative hematoxylin and eosin stained paraffin liver section of diabetic rats (**a**) shows sinusoidal congestion (arrows), the bloated hepatocytes with vacuolar degeneration (arrowhead) and necrosis of the hepatocytes (mainly around the central vein, dashed arrows). Liver of diabetic rats treated with MF (**b**) depicts areas of micronecrosis (arrows) and hepatocytes with vacuolar degeneration (dashed arrows). In liver of diabetic rats treated with non cross-linked microparticles formulation (**c**), a slight congestion (arrows) and vacuolization of the hepatocytes cytoplasm with a low intensity (dashed arrows) is seen. Liver of diabetic rats treated with cross-linked microparticles formulation CH1M (**d**) shows normal structure of hepatocytes with only a few degraded cells (arrows). Magnification ×200.

Diabetes is the most common cause of liver diseases, which include non-alcoholic fatty liver disease, cirrhosis, hepatocellular carcinoma and acute liver failure [46]. Thus, the liver morphology was examined, as shown in Figure 8a–d. On microscopic examination, histological changes were seen in the liver of rats with diabetes. The lesions were characterized by engorgement of blood vessels along with sinusoidal hemorrhages, degeneration, necrosis and cellular changes (increased cytoplasmic eosinophilic granularity, vacuolar degeneration) (Figure 8a). In the liver of diabetic rats treated with MF, damage of the trabecular structure of the organ with micronecrosis and areas with vacuolar degeneration were observed (Figure 8b). Interestingly, in liver of rats treated with both non cross-linked and cross-linked microparticles, properly preserved trabecular structure and lobules of the liver were recorded. A slight vacuolization of the cells cytoplasm, significantly reduction in sinusoidal congestion and only a small areas of necrosis were observed (Figure 8c,d).

**Figure 9:**
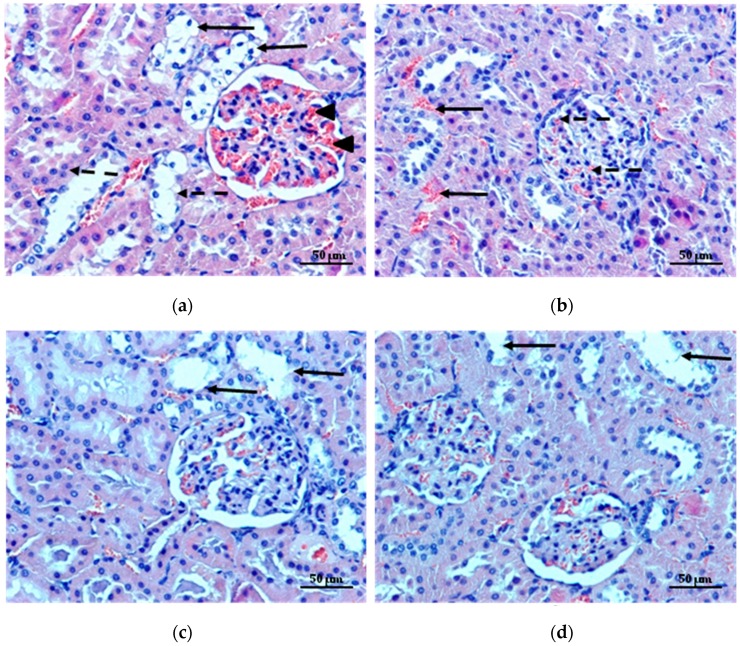
Representative hematoxylin and eosin stained paraffin kidney section of diabetic rats (**a**) shows severe cellular injury: swelling (arrows), vacuolization and detachment of epithelial cells from the basement membrane (dashed arrows), strong congestion of glomeruli (arrowheads). Kidney of diabetic rats treated with MF (**b**) shows improvement in the renal histology, reduction in congestion in the parenchyma (arrows) and reduction in renal glomeruli (dashed arrows). Kidney sections of diabetic rats treated with non cross-linked microparticles formulation C (**c**) and cross-linked microparticles formulation CH1M (**d**) depict a significant improvement in the structure of the organ and reduction of lesions. Normal architecture, without congestion, with only slight changes in renal tubular epithelial cells is visible (arrows). Magnification ×200.

Diabetic nephropathy is one of the most common complications of diabetes, which might provide the ultimate loss of renal functions [47]. The effects of microparticles on the diabetic rats kidneys are shown in Figure 9a–d. In the kidneys of diabetic rats, tubular damage and glomerular lesion were observed (Figure 9a). Pathological changes in the tubules were manifested by swelling, vacuolization and detachment of epithelial cells from the basement membrane (Figure 9a). MF administration to diabetic rats improved the renal histology, congestion in the parenchyma and renal glomeruli, and various staining of cells cytoplasm were not observed (Figure 9b). A microscopic examination of the kidneys of rats treated with non cross-linked and cross-linked microparticles showed a significant improvement in the structure of the organ and reduction of lesions compared to the animals untreated (Figure 9c,d).

Histopathological studies indicated that both, non cross-linked microparticles formulation C and chitosan cross-linked ALG microparticles formulation CH1M reduced the degenerative changes of pancreas, liver and kidneys. Additionally, histopathological studies of examined tissues of diabetic rats treated with ALG microparticles placebo showed a normal structure with no degenerative changes in organs (data not shown). Moreover, the animals neither produced any signs of toxicity nor mortality symptoms, and no changes in their behavior and body weight was observed, which indicates the non-toxic nature of designed microparticles.

## 3. Materials and Methods

### 3.1. Materials

Metformin hydrochloride (MF) was obtained from Debao Fine Chemical Co. (Zhengzhou, China). Sodium alginate (ALG) low viscosity (2%, 132.6 mPa∙s for 2% solution at 25 °C; molecular weight about 147,000; 61% mannuronic acid and 39% guluronic acid), streptozotocin, mucin type II and gelatin type B from bovine skin and carboxymethyl cellulose sodium salt were purchased from Sigma Aldrich (Steinheim, Germany). High quality medium molecular weight (MMW 200–400 kDa; viscosity 370–395 mPa∙s at 25 °C, 1% in 1% acetic acid) chitosan with deacetylation degree 79.9% (determined by titration method [48]) was obtained from Heppe Medical Chitosan GmbH (Haale, Germany). Potassium dihydrogen phosphate, sodium hydroxide, hydrochloric acid, methanol, propane-1,2-diol, acetonitrile, calcium chloride, trichloroacetic acid were obtained from Chempur (Piekary Śląskie, Poland). Water was distilled and passed through a reverse osmosis system Milli-Q Reagent Water System (Merck Millipore, Billerica, MA, USA). Porcine stomach mucosa from large white pigs weighting ≈200 kg was obtained from the veterinary service (Turośń Kościelna, Poland). Samples were stored at −20 °C and before the experiment were defrosted and cut into 5 mm in diameter and 2 mm thick pieces. Commercially available non modified tablets with MF (Metformax 500 mg; TEVA UK Ltd., batch no.: 16115015, expiry date: 03.2018) were purchased locally and used as a control.

### 3.2. Preparation of Chitosan Cross-Linked ALG Microparticles by Spray Drying

The composition of designed chitosan cross-linked ALG microparticles was presented in Table 6. Based on the preliminary studies, 2% (*w/w*) ALG solution and MF:ALG ratio 2:1 were used [25]. As cross-linking agent, 0.5%, 0.1% and 0.05% (*w/w*) chitosan solutions in 1% acetic acid were applied. The polymers solutions were adjusted to pH 5.0—ALG solution using HCl and chitosan using NaOH. Chitosan solutions were added dropwise into the ALG solution and stirred until homogenous mixture appeared using homogenizer (Heidolph, Schwabach, Germany). Viscosity of obtained solutions was determined using a rotational viscometer (Viscotester E Plus—Thermo Haake, Thermo Electron Corporation, Karlsruhe, Germany). To obtain chitosan cross-linked ALG microparticles, 2% ALG solution and 0.05% and 0.1% chitosan were selected to spray drying using Mini Spray Dryer B-290 (Büchi, Flawil, Switzerland). Mixture of 2% ALG solution cross-linked by 0.5% chitosan was characterized by high viscosity (2316.4 ± 11.4 mPa∙s), therefore its spray drying was limited. The parameters of the process were chosen after a number of preliminary studies and included: flow rate 4.5 mL/min, relative spray rate 37 m^3^/h and spray flow 600 L/h. The inlet and outlet temperatures were established at 130 °C and 76 °C, respectively. Non cross-linked ALG microparticles obtained by spray drying of 2% ALG solution and 2:1 MF:ALG ratio were used as a control in the study (formulation C) [25].

### 3.3. Characteristics of Microparticles

#### 3.3.1. Morphology and Size

Microparticles were analyzed by optical microscope (Motic BA400, Wetzlar, Germany) and by scanning electron microscope (SEM) (Hitachi S4200, Tokyo, Japan). Before imaging samples were sputter-coated with gold in an argon atmosphere (Leica EM AC 2000, Wetzlar, Germany). The microparticles size distribution and polydispersity index were studied using Zetasizer NanoZS90 (Malvern Instruments, Malvern, UK) by laser light-diffraction technique after suspending in propane-1,2-diol (propane-1,2-diol was used because in aqueous medium swelling and dissolving of microparticles was observed).

#### 3.3.2. HPLC Analysis

The concentration of MF in the medium was determined by the HPLC system Agilent Technologies 1200 equipped with a G1312A binary pump, a G1316A thermostat, a G1379B degasser and a G1315B diode array detector (Agilent, Waldbronn, Germany). Data collection and analysis were performed using Chemstation 6.0 software (Agilent). Isocratic separation was achieved on Waters Spherisorb^®^ 5.0 μM ODS 4.6 × 250 mm, 5 μm column (Waters Corporation, Milford, CT, USA). Mobile phase was acetonitrile:methanol:phosphate buffer pH 3.0 (20:20:60, *v*/*v*), the flow rate was 1.0 mL/min and UV detection was performed at a wavelength of 240 nm [25]. The column temperature was maintained at 25 °C. For injection into the HPLC system, 20 µL of sample was used. All reagents used for analysis were HPLC grade. The retention time of MF was 2.8 min. Standard calibration curve was linear over the range of 1–100 μg/mL with the correlation coefficient (R^2^) 0.999.

#### 3.3.3. MF Loading and Percentage Yield

MF loading in the microparticles was determined by dissolving an accurately weighed amount of microparticles (20 mg) in 10 mL of phosphate buffer pH 6.8 and agitating for 24 h at 150 rpm in a water bath [25]. The sample solution was further diluted, filtered through 0.45 µm cellulose acetate Millipore filters (Merck Millipore, Billerica, MA, USA) and filtrate was analyzed by HPLC method (as described in the point 3.3.2. HPLC analysis). The percentage yield of MF in the ALG microparticles was determined by using the formula:

L = Q_m_/W_m_ × 100, (1)

where L is the percentage of drug loading, Q_m_ is the drug loaded in the microparticles and W_m_ is the weight of the microparticles.

The mean drug encapsulation efficiency was calculated by the equation:

EE = Q_a_/Q_t_ × 100, (2)

where EE is the percentage of encapsulation efficiency, Q_a_ is the actual drug content, and Q_t_ is the theoretical drug content.

Percentage production yield was calculated as the relationship of the achieved weight of the microparticles related to the entire amount of the theoretical weight of drug and polymer:

Y = W_m_/W_t_ × 100, (3)

where Y is the percentage production yield, W_m_ is the weight of microparticles and W_t_ is the theoretical weight of drug and polymer.

#### 3.3.4. Zeta Potential

Zeta potential measurements were performed using a Zetasizer NanoZS90 (Malvern Instruments, Malvern, UK). Before measurements, microparticles were suspended in propane-1,2-diol. Data were obtained from Zetasizer Software 6.20 (Malvern Instruments, Malvern, UK).

#### 3.3.5. Swelling Properties

Swelling properties were evaluated by placing microparticles (200 mg) in beakers containing 25 mL of 0.1 M HCl (pH 1.2) and stirring at 100 rpm at 37 ± 1 °C in the water bath. At predetermined time interval, medium was carefully removed by filtration and swollen microparticles were weighed. Test was performed until a constant weight of microparticles was obtained [49]. The swelling ratio was calculated by using the following formula:

SR = (W_S_ − W_0_)/W_0_ × 100, (4)

where SR is the swelling ratio, W_0_ is the initial weight of microparticles and W_S_ is the weight of microparticles after swelling.

#### 3.3.6. Mucoadhesiveness

Evaluation of mucoadhesiveness was performed using TA.XT.Plus Texture Analyzer (Stable Micro Systems, Godalming, UK) and three different models of mucoadhesive material: gelatin disc, mucin gel and porcine stomach mucosa. Experimental parameters of the process were chosen during preliminary tests and set as follows: pretest speed 0.5 mm/s, test speed 0.1 m/s, contact time 180 s, post test 0.1 mm/s, applied force 1 N. Gelatin discs were prepared by pouring 30% (*w*/*w*) aqueous solution into a Petri dish. Layer of mucin was prepared by absorbing 10% mucin gel on discs with cellulose fiber (5 mm in diameter). The tests were conducted at 37 ± 1 °C. Adhesive layers were adhered to an upper probe and moisturized (excepted mucin) with 0.1 M HCl (pH 1.2) [34]. The mucoadhesive properties were determined as the maximum detachment force (F_max_) and the work of mucoadhesion (W_ad_)—calculated from the area under the force versus distance curve, expressed in µJ.

### 3.4. In Vitro MF Release

Due to the flotation of microparticles, for the in vitro MF release test, apparatus type I (Erweka Dissolution tester type DT 600HH, Heusenstamm, Germany) was used [50]. Microparticles were placed in the basket, immersed in 500 mL of 0.1 M HCl (pH 1.2) and stirred at 50 rpm. In each study, the amount of microparticles equivalent to 500 mg of MF was analyzed. Samples were withdrawn and filtered through 0.45 µm cellulose acetate Millipore filters (Merck Millipore, Billerica, MA, USA) at predetermined time intervals and replaced with fresh dissolution medium. During the dissolution process, the temperature was maintained at 37 ± 1 °C. The amount of released MF was analyzed by HPLC method (as described in the point 3.3.2. HPLC analysis).

### 3.5. Mathematical Modeling of MF Release Profile

MF release data were analyzed according to zero order kinetic, first order kinetic, Higuchi model, Korsmeyer–Peppas equation and Hixson–Crowell cube root law to characterize mechanism of the drug release. The constants of release kinetics and the regression coefficients (R^2^) were calculated from the slope of plots by linear regression analysis.

Zero order kinetic describes formula:

F = k × t,(5)

First order kinetic describes formula:

lnF = k × t,(6)

Higuchi model describes equation:

(7) $$F=k\sqrt{t},$$

Korsmeyer–Peppas model is expressed by the following equation:

F = kt^n^, (8)

Hixson–Crowell model:

1 − (1 − F)^1/3^ = kt, (9)

where F is the fraction of drug release, k is the the release constant and t is the the time. For the Korsmeyer–Peppas model, the fraction of drug remaining at time t was determined for every time interval log (Mt/M∞) and plotted against the log of time t. The slope of the line was taken as the value of *n*, diffusional release exponent used to interpretation of release mechanism [35,51].

### 3.6. Stability Studies

The physical change, mean diameter, Zeta potential, drug loading, mucoadhesion and MF release profiles from non cross-linked microparticles formulation C and cross-linked microparticles formulations CH1M and CH2M were assessed after 6 months of storage at 25 ± 2 °C and RH 60% ± 5% and 40 ± 2 °C and RH 75% ± 5%.

### 3.7. Differential Scanning Calorimetry (DSC)

DSC analysis of MF, ALG, chitosan, non cross-linked microparticles formulation C, cross-linked microparticles placebo CH1P and cross-linked microparticles formulation CH1M (with high drug loading and optimal release profile) was performed using an automatic thermal analyzer system (DSC TEQ2000, TA Instruments, New Castle, DE, USA). Each sample was precisely weighed (5 mg) and placed in sealed aluminum pan. An empty pan sealed was used as a reference. Temperature calibrations were performed using indium and zinc as standard. Samples were heated from 25 °C to 280 °C at scanning rate of 10 °C/min under nitrogen flow of 20 mL/min [52].

### 3.8. In Vivo Studies

#### 3.8.1. Animals

The in vivo experimental protocol was approved by Local Committee on the Ethical Use of Animals of the Medical University in Białystok, Poland, number 156/2015. Four-week old healthy male albino rats outbred Wistar Cmdb:Wi weighing 150–200 g were obtained from Center for Experimental Medicine (Białystok, Poland). The animals were housed in individual plastic cages, two animals per cage, in a room maintained at 22 ± 2 °C, air humidity 35%–60% with a 12 h light-dark cycle (8.00–20.00). All experiments were carried out in a quiet, diffusely lit room. The animals were acclimatized to the laboratory environment for 1 week before commencement of the experiment. Animals were fed with high fat rat feed, ordered from the Ssniff Spezialdiäten GmbH (Soest, The Netherlands) consisting of 15% fat, 49% carbohydrate and 36% protein with total calorific value 3646 kcal/kg. Food and water were freely available.

#### 3.8.2. Experimental Design

Zhang et al. reports that the combination of high fat diet and multiple injections of streptozotocin at low doses (30 mg/kg b.wt. weekly for 2 weeks) in rats provide a stable animal model of type 2 diabetes, which imitates clinical situation in humans [39]. To in vivo tests, chitosan cross-linked ALG microparticles formulation CH1M was selected due to its optimal parameters evaluated in vitro. After 1-week acclimatization, Wistar rats were fed with high fat rat feed with free access to water for four weeks. Diabetes was induced by a double with a one-week interval intraperitoneal (i.p.) streptozotocin injection of 30 mg/kg b.wt. dissolved immediately before administration in citrate buffer (pH 4.5) [39]. Two weeks after the first injection, glucose level was measured through blood collection from the tip of tail of the animals after 12 h overnight starving with free access to water. Blood glucose measurement was performed using a glucometer (OneTouch Select, LifeScan, Milpitas, CA, USA). Animals with plasma glucose concentration above 200 mg/dL were considered as diabetic and were used in experimental work. Wistar rats were divided into groups consisting of six animals. Group I, animals treated with 0.5% carboxymethyl cellulose sodium salt (control group); Group II, non cross-linked ALG microparticles placebo; Group III, non cross-linked microparticles formulation C; Group IV, cross-linked microparticles formulation CH1M; and Group V, commercially available non-modified tablet with MF. Non cross-linked microparticles formulation C, cross-linked microparticles formulation CH1M and powdered commercially available non-modified tablet with MF were suspended in 0.5% carboxymethyl cellulose sodium salt and administered in an amount corresponding to 100 mg/kg b.wt. of MF, by gastric intubation with a needle gauge in a final volume of 1 mL every day for 28 days. For signs of microparticles toxicity, behavioral changes and body weight of animals treated with non cross-linked ALG microparticles placebo, non cross-linked microparticles formulation C and cross-linked microparticles formulation CH1M, were also monitored weekly throughout the experimental period. Care of the animals and all experimental steps were carried out according to the experimental protocol of the Local Committee on the Ethical Use of Animals of the Medical University in Białystok, Poland.

#### 3.8.3. Blood Glucose Levels

Blood was collected from the tip of tail of the animals after 12 h overnight fasting and blood glucose measurement was performed using a glucometer (OneTouch Select, LifeScan, Milpitas, CA, USA). The blood glucose levels were monitored at 3, 6, 9, 12, 15, 18, 21, 24 and 28 days of treatment. Testing intervals were recommended by the Local Committee on the Ethical Use of Animals of the Medical University in Białystok to regenerate tip of rats tails. The obtained data were plotted against time and expressed as percentage glucose reduction in blood.

#### 3.8.4. MF Pharmacokinetic Parameters

After 28 days treatment, there was a one-week break in drug administration. Afterwards, non cross-linked microparticles formulation C, cross-linked microparticles formulation CH1M and commercially available non-modified tablet were single administered intragastric in an amount corresponding to 100 mg/kg b.wt. of MF. Rats were anesthetized using pentobarbital (40 mg/kg b.wt., i.p.) and blood samples were collected from the heart after 1 h, 2 h, 4 h, 6 h, 8 h and 10 h after administration of non cross-linked microparticles formulation C, cross-linked microparticles formulation CH1M and commercially available non-modified tablet. The blood was collected in tubes with 1 mL 3.8% (*w*/*v*) citrate buffer. For the plasma separation, blood was centrifuged for 15 min at 2000 rpm at 4 °C (Centrifuge 5810R Eppendorf AG, Hamburg, Germany). Plasma samples were stored at −70 °C until measurement. Before determination of MF concentration, blood plasma was deproteinized by using 6% (*w*/*v*) trichloroacetic acid, centrifuged at 3500 rpm for 10 min (Centrifuge MPW-223e, MPW Med. Instruments, Warsaw, Poland) and supernatant was filtered through 0.22 µm nylon filters (Merck Millipore, Billerica, MA, USA) [53]. Twenty microliters of deproteinized plasma was injected directly into HPLC column for analysis (as described in the point 3.3.2. HPLC analysis). The retention time of MF was approximately 4 min. Based on the obtained data of plasma MF concentration, pharmacokinetic parameters (AUC, T_max_ and C_max_) were calculated using GraphPad 5.0 software (GraphPad, San Diego, CA, USA).

#### 3.8.5. Histopathological Studies

At the end of the study, animals were sacrificed and liver, pancreas and kidney were instantly dissected out. Collected organs were fixed in 10% formalin and routinely embedded in paraffin for histopathological examinations. The fixed tissues were then cut into small pieces and put in a labelled tissue cassette for dehydration process (Leica, ASP 300, Nussloch, Germany). Sections, 4 μm thick, were cut using rotary microtome Leica 2025 (Leica Microsystems, Wetzlar, Germany) and stained with hematoxylin (H) and eosin (E) or with Azan method for general histological examination. Tissue sections were passed through series of alcohol for dehydration (70%, 90% and 100%) and examined using a light microscope Olympus BX41 (Olympus Corporation, Tokyo, Japan) at 200× magnifications interfaced with an image analysis system. The images were captured using a DP12 camera (Olympus Corporation, Tokyo, Japan) [54,55].

### 3.9. Statistical Analysis

Results were analyzed using Statistica 10.0 software (StatSoft, Tulsa, OK, USA). Quantity variables were expressed as the mean and standard deviation. Statistical analysis were performed using one-way analysis of variance (ANOVA) or Kruskal–Wallis test. Differences between groups were considered to be significant at *p* < 0.05.

## 4. Conclusions

The obtained data suggest that the spray-drying technique might be used to prepare chitosan cross-linked ALG microparticles with MF using 2% ALG solution and 0.1% or 0.05% chitosan as cross-linking agent. Designed microparticles were characterized by spherical shape with slightly rough area and beneficial swelling and mucoadhesive properties. Studies in vitro and in vivo showed that chitosan cross-linked ALG microparticles provided sustained MF release. Microparticles also exhibited good pharmacokinetic parameters (lower C_max_ and higher t_max_ compared to commercially available tablet with MF). Histopathological analysis of pancreas, liver and kidney of diabetic rats showed reduction in degenerative changes. Moreover, the obtained data have shown that modification of ALG structure by chitosan cross-linking is characterized by no toxicity effect after oral administration.

## Figures and Tables

**Table 1:** Characteristics of non cross-linked microparticles formulation C and cross-linked microparticles formulation CH1M and CH2M.

Formulation	Zeta Potential (mV)	Production Yield (%)	Encapsulation Efficiency (%)	Percent Loading (%)	Mean Diameter (µm)	Polydispersity Index
C	−1.3 ± 0.7	61.7 ± 2.1	113.4 ± 2.3	75.6 ± 1.5	3.0 ± 1.6	0.38 ± 0.1
CH1M	6.7 ± 1.3	61.8 ± 1.5	94.1 ± 3.2	79.9 ± 1.6	2.3 ± 1.1	0.52 ± 0.2
CH2M	5.2 ± 0.3	85.9 ± 1.4	95.4 ± 1.9	76.5 ± 2.2	3.2 ± 1.7	0.42 ± 0.2

**Table 2:** Mucoadhesive properties of non cross-linked microparticles C and cross-linked microparticles CH1P, CH2P, CH1M, and CH2M (*n* = 6).

Formulation	Type of Adhesive Layer					
	Gelatin		Mucin		Porcine Stomach Mucosa	
	F_max_ (N) *	W_ad_ (µJ) **	F_max_ (N) *	W_ad_ (µJ) **	F_max_ (N) *	W_ad_ (µJ) **
C	0.5 ± 0.2	283.3 ± 50.6	0.6 ± 0.3	342.3 ± 29.1	0.6 ± 0.2	467.5 ± 17.9
CH1P	0.5 ± 0.1	314.8 ± 13.9	0.8 ± 0.2	384.7 ± 29.7	1.4 ± 0.2	782.6 ± 29.2
CH2P	0.5 ± 0.2	312.4 ± 21.2	0.7 ± 0.2	381.2 ± 13.1	1.3 ± 0.2	564.4 ± 18.6
CH1M	0.5 ± 0.1	310.1 ± 16.8	0.8 ± 0.3	378.7 ± 35.5	1.2 ± 0.1	779.5 ± 24.8
CH2M	0.6 ± 0.2	293.6 ± 40.4	0.7 ± 0.1	349.4 ± 31.3	1.2 ± 0.2	549.1 ± 23.7

* Maximum detachment force; ** Work of adhesion.

**Table 3:** Models of MF release from non cross-linked microparticles formulations C and cross-linked microparticles formulations CH1M, CH2M.

Formulation	Zero Order Kinetics		First Order Kinetics		Highuchi Model		Korsmeyer–Peppas Model			Hixson–Crowell Model	
	R^2^	K	R^2^	K	R^2^	K	R^2^	K	*n*	R^2^	K
C	0.52	4.62	0.73	0.21	0.66	18.76	0.59	0.28	0.12	0.65	0.19
CH1M	0.71	6.27	0.97	0.18	0.85	31.55	0.76	0.27	0.11	0.93	0.57
CH2M	0.87	6.69	0.95	0.22	0.96	25.03	0.90	0.32	0.19	0.94	0.22

R^2^: correlation coefficient; K: release constant; and *n*: the release exponent.

**Table 4:** Stability characteristics of non cross-linked microparticles formulation C and cross-linked microparticles formulations CH1M and CH2M after 6 months storage at 40 ± 2 °C and 75% ± 5% RH.

Evaluation Parameter		Formulation		
		C	CH1M	CH2M
Mean diameter (µm)		2.7 ± 1.3	2.1 ± 1.5	2.9 ± 1.4
Percent drug loading (%)		73.1 ± 5.7	73.6 ± 4.3	70.7 ± 1.4
Zeta potential (mV)		−1.16 ± 0.3	5.9 ± 1.4	4.8 ± 0.6
Mucoadhesion to:				
Gelatin disc	F_max_ (N) *	0.4 ± 0.1	0.4 ± 0.1	0.3 ± 0.1
	W_ad_ (µJ) **	288.2 ± 18.8	304.8 ± 26.8	233.6 ± 19.3
Mucin gel	F_max_ (N) *	0.5 ± 0.2	0.5 ± 0.1	0.5 ± 0.2
	W_ad_ (µJ) **	316.8 ± 17.9	318.6 ± 21.7	323.2 ± 26.7
Porcine stomach mucosa	F_max_ (N) *	0.4 ± 0.1	0.8 ± 0.2	0.8 ± 0.2
	W_ad_ (µJ) **	379.7 ± 36.2	715.9 ± 31.6	456.1 ± 34.6

* Maximum detachment force; ** Work of adhesion.

**Table 5:** Pharmacokinetic parameters of metformin hydrochloride plasma concentration after single oral administration of non cross-linked microparticles formulation (C), cross-linked microparticles formulation (CH1M) and commercially available tablet with metformin hydrochloride (MF) in an amount corresponding to the 100 mg/kg b.wt. (*n* = 6).

Formulation	C_max_ (μg/mL)	t_max_ (h)	AUC_0→10_ (μg·h/mL)
C	3.03 ± 0.70	6.0	30.51 ± 0.51
CH1M	2.37 ± 0.34	6.0	22.10 ± 0.38
MF	8.13 ± 0.50	2.0	23.43 ± 0.78

**Table 6:** Composition of chitosan cross-linked ALG microparticles.

Formulation	Concentration of ALG Solution (*w*/*w*)	Concentration of Chitosan Solution (*w*/*w*)	MF:ALG Ratio
C	2%	-	2:1
CH1P	2%	0.10	-
CH2P	2%	0.05	-
CH1M	2%	0.10	2:1
CH2M	2%	0.05	2:1
